# Virtual Reality Analgesia During Venipuncture in Pediatric Patients With Onco-Hematological Diseases

**DOI:** 10.3389/fpsyg.2018.02508

**Published:** 2018-12-20

**Authors:** Barbara Atzori, Hunter G. Hoffman, Laura Vagnoli, David R. Patterson, Wadee Alhalabi, Andrea Messeri, Rosapia Lauro Grotto

**Affiliations:** ^1^Department of Health Sciences, University of Florence, Florence, Italy; ^2^Department of Mechanical Engineering, University of Washington, Seattle, WA, United States; ^3^Pediatric Hospital’s Psychology, Meyer Children’s Hospital, Florence, Italy; ^4^Department of Rehabilitation Medicine, University of Washington, Seattle, WA, United States; ^5^Department of Computer Science, King Abdulaziz University, Jeddah, Saudi Arabia; ^6^Department of Computer Science, Effat University, Jeddah, Saudi Arabia; ^7^Pain Service and Palliative Care, Meyer Children’s Hospital, Florence, Italy; ^8^Multidisciplinary Analysis of Relationship in Health Care (MARHC) Lab, Pistoia, Italy

**Keywords:** virtual reality, children, adolescents, pain, pediatric cancer, distraction

## Abstract

**Background:** Venipuncture is described by children as one of the most painful and frightening medical procedures.

**Objective:** To evaluate the effectiveness of Virtual Reality (VR) as a distraction technique to help control pain in children and adolescents undergoing venipuncture.

**Methods:** Using a within-subjects design, fifteen patients (mean age 10.92, *SD* = 2.64) suffering from oncological or hematological diseases received one venipuncture with “No VR” and one venipuncture with “Yes VR” on two separate days (treatment order randomized). “Time spent thinking about pain”, “Pain Unpleasantness”, “Worst pain” the quality of VR experience, fun during the venipuncture and nausea were measured.

**Results:** During VR, patients reported significant reductions in “Time spent thinking about pain,” “Pain unpleasantness,” and “Worst pain”. Patients also reported significantly more fun during VR, and reported a “Strong sense of going inside the computer-generated world” during VR. No side effects were reported.

**Conclusion:** VR can be considered an effective distraction technique for children and adolescents’ pain management during venipuncture. Moreover, VR may elicit positive emotions, more than traditional distraction techniques. This could help patients cope with venipuncture in a non-stressful manner. Additional research and development is needed.

## Introduction

For many children, venipuncture is one of the most frightening aspects of visiting a hospital (Duff, 2003; Caprilli and Messeri, 2006). Experiencing pain and anxiety during medical procedures can result in several negative consequences, such as higher levels of fear. Unpleasant early medical experiences can affect patients’ perception of healthcare, can increase pain and suffering during subsequent medical visits, and can reduce preventative healthcare, affecting lifelong health (El-Housseiny et al., 2014). Developing expectations of pain (e.g., via memories for previous painful medical procedure experiences, Noel et al., 2015) can increase how much pain patientsexperience during a procedure, via top-down amplification of neural signals coming into the brain from the pain receptors (Fields, 2018). For patients with chronic diseases, venipuncture can be particularly painful and stressful (Bisogni et al., 2014). Adequate pain management is especially important for patients who receive multiple venipunctures. Indeed, untreated pain can have damaging effects on future pain perceptions and can provoke negative psychological effects (Weisman et al., 1998) and a simple procedure, such as venipuncture, could represent an additional stressor in an already critical condition.

Traditional distraction techniques (e.g., reading books or listening to music) are some of the most common psychological strategies for the reduction of procedural pain and anticipatory anxiety during venipuncture (Birnie et al., 2018).

Birnie et al. (2018) conducted a Cochrane Review of Psychological interventions for needle-related procedural pain and distress in children and adolescents (5550 participants). The studies evaluated in Birnie et al’s (2018) review included venipuncture, intravenous insertion, and vaccine injections in patients aged two to 19 years. The most common psychological interventions were distraction (*n* = 32 studies) and only two VR distraction studies were included in Birnie et al’s Cochrane review.

Virtual Reality (VR) is showing promise as an innovative distraction technique for pain management among children undergoing medical procedures (Hoffman, 1998; Hoffman et al., 2000a; Bailey and Bailenson, 2017; Atzori et al., 2018b,c). VR reduces the cognitive component of pain (time spent thinking about pain), but also reduces the affective component (pain unpleasantness) and the sensory component (worst pain), as consistently shown in studies with adult and pediatric participants with burn injuries (Hoffman, 1998; Hoffman et al., 2000a,b, 2011; Atzori et al., 2018a; Soltani et al., 2018). Unlike traditional distractions, VR allows the user to be immersed in a computer-generated environment, wearing a Head Mounted Display (HMD), or similar goggles, that occlude the patient’s view of the hospital treatment room and blocks sounds of the real environment (Hoffman, 2004). The user can also interact with the VR environment, if the software allows it (Hoffman et al., 2006; Won et al., 2017). Although the mechanism(s) of how VR reduces pain are still under investigation, Hoffman and colleagues applied the Eccleston and Crombez (1999) Attention Pain Theory to explain how VR can reduce the perception of pain. Attention is required to feel pain, but the illusion of being in a virtual environment and the patients’ interaction with the objects in the virtual world reduce the amount of attentional resources the patient’s brain has available to attend to the painful stimulus, thus reducing conscious pain perception (Hoffman et al., 2004a). Much of VR’s therapeutic power is derived from its ability to divert attention away from painful medical interventions. Moreover, in addition to reducing pain, patients report having fun during burn wound care when playing VR (Hoffman et al., 2004b). The immersiveness of the VR systems, such as the quality of the helmet, and the patients’ ability to interact with objects in the virtual world, influence how much VR reduces pain in adults (Hoffman et al., 2004c, 2006; Wender et al., 2009). Moreover, as converging objective evidence, fMRI brain scan studies have shown reduced activity of the brain areas involved in pain perception in healthy adult volunteers distracted with VR during a brief painful thermal stimulus (Hoffman et al., 2004b, 2007).

There is a growing interest in using VR for distraction among children and adolescents; however, to date most clinical studies on VR analgesia have included burn patients during physical therapy (Carrougher et al., 2009) or during burn wound cleaning (Das et al., 2005; Maani et al., 2011; Hoffman et al., 2014; Dascal et al., 2017). VR has also emerged as a useful intervention for procedural pain in patients suffering from chronic diseases, such as cancer patients’ support during medical treatments (Chirico et al., 2016) and pain management during invasive procedures in pediatric cancer patients (Wint et al., 2002; Gershon et al., 2003, 2004; Wolitzky et al., 2005). Results exploring the use of VR distraction during needle related procedures have been encouraging, but mixed. Several studies have shown the predicted pattern, but non-significant reductions in patients’ pain during painful cancer treatment procedures for children. For example, in a non-immersive VR study by Sanders Wint et al. (2002), patients watched a traditional movie via see-through glasses, and found no significant reduction in cancer patients’ ratings of pain during venipuncture. A small early study using a relatively low tech VR goggles, did not find significant reduce patients pain during IV placement (Gold et al., 2006). Similarly, using early low tech VR technology, although they found some encouraging patterns, Gershon et al. (2004) found no significant reduction in patients’ pain during port placement. Due in part to recent dramatic increases in the availability of immersive VR equipment (e.g., Hoffman et al., 2014), there is growing interest in using VR as a non-pharmacologic analgesic. Gold and Mahrer (2017) recently published a large definitive clinical study exploring the use of immersive VR using untethered Oculus VR goggles, and found significant reductions in pain during blood draws. Using tethered Oculus VR DK2 goggles, Piskorz and Czub (2018) found significant reductions in pain during blood draws in children with kidney problems.

The aim of the present study was to investigate VR effectiveness as a distraction technique for pain management in children and adolescents with onco-hematological diseases undergoing venipuncture. Based on the Eccleston and Crombez (1999) interpretative model, we predicted that patients would focus their attentional resources on VR, and would have less attentional resources available to process incoming pain signals, with the result of reduced pain perception during venipuncture. We expected that patients interacting with VR during venipuncture would report less pain (“pain unpleasantness”, “time spent thinking about pain” and “worst pain”) compared to “treatment as usual”. The current study is the first to measure how much fun patients experienced during venipuncture, during No VR vs. during Yes VR. We predicted that patients would report significantly higher levels of fun during VR (a surrogate measure of positive affect), compared with the standard care (no VR), without side effects (Sharar et al., 2016).

## Materials and Methods

### Participants

From February 2014 to July 2016, patients attending the Service of Pediatric Oncology and Hematological Diseases of an Italian Children’s hospital participated. Children and adolescents who needed to undergo venipuncture twice in a year, for intravenous placement during chemotherapy, transfusions, magnetic resonance or blood analysis were recruited. Patients were selected according to the following criteria based on the existent literature (Won et al., 2017; Atzori et al., 2018a): children and adolescents who were able to understand Italian language, complete the tests, wear the helmet and interact with the VR environment, without any physical or psychological impairments. Patients with a venous access already inserted, with a diagnosis of epilepsy, not accompanied by their legal guardians, older than 17 years old and younger than 7 years old were excluded. Moreover, patients who wanted their own distraction tool (i.e., a book, a videogame or mp3-player) during the venipuncture, were excluded.

Seventeen patients met the inclusion criteria. However, one of them withdrew because he decided to use his own distraction technique and another patient withdrew because he didn’t want to use VR during the second venipuncture (the reason was not indicated). A total of 15 patients (66.7% males, 33.3% females; mean age 10.92, *SD* = 2.64, see Table 1) took part in the study. All patients had previously received at least one venipuncture by nurses of the Service of Pediatric Oncology and Hematological Diseases and none of patients was at the first access. No patient reported pain before the beginning of the procedure. No patient had previously used VR before the study and all participants were familiar with the wireless mouse. All patients underwent two venipunctures on two different days: one venipuncture with No VR, and one venipuncture with Yes VR on a second visit (treatment order randomized). The mean time between the first and the second venipuncture was 26.6 days (± 24.5).

**Table 1:** Demographic, clinical and procedural characteristics.

Participants’ characteristics (*n* = 15)	Mean (SD)	
**Age**	10.92 (2.64)	
	***n***	**%**
**Sex**		
Male	10	66.7
Female	5	33.3
**Disease**		
Cancer	11	73.3
Blood diseases	4	26.7
**Italian origins**		
Yes	11	73.3
No	4	26.7
**Painful procedure (*n* = 30)**		
Blood draw	25	83
Venous access	5	17

### Procedure

This research was conducted in accordance with the Declaration of the World Medical Association^1^. The protocol was accepted by the Ethical Committee of the Hospital and the study was approved by the physicians and the nurses of the Service of Pediatric Oncology and Hematological Diseases and conducted in collaboration with the Pediatric Psychology Service and the Pain Therapy Service. Patients meeting the inclusion criteria were approached in the waiting room by a psychologist before the procedure in order to inform the families and get the signed written informed consent form by the patient’s caregivers. The written informed consent was obtained from all the parents of the participants. All participants provided written informed consent/assent in accordance with the Declaration of Helsinki. The study was described to the parents/guardians. If the parent/guardian’s gave permission, the research team then explained the study to the child in age-appropriate language, to see if the child was willing to participate in the study. Both children and their parents were encouraged to ask questions. Before the procedure began, patients and their caregivers were next escorted to the treatment room, and then the nurse arrived.

Using a within-subjects design, patients were assigned to the control condition (“No VR”) or the experimental condition (“Yes VR”) (treatment order randomized). Patients underwent the second venipuncture using the distraction technique not used the first time. The “No VR” control condition consisted of non-medical conversation by the nurse who performed the venipuncture (standard of care). In the “Yes VR” condition, patients interacted with VR during venipuncture. Before the nurse arrived, patients had 5 min to learn how to use the VR system. The helmet and the earphones included in the VR system were worn at the arrival of the nurse for the procedure and removed after the procedure. In both conditions, during the venipuncture, the patient, a nurse, the patient’s parent/caregiver and the psychologist researcher were present. After the procedure, the nurse left the room and patients completed the self-report questionnaire (pain ratings). No reward was given to patients for participating.

### Measures

At the end of the procedure, patients filled out a brief self-report questionnaire aimed to evaluate the pain, the quality of the VR experience, nausea and fun. Patients responded by giving a 0–10 score on a horizontal Visual Analogue Scale (VAS; Price et al., 1994; Bailey et al., 2012). Pain, evaluated in its cognitive component (time spent thinking about pain), affective component (pain unpleasantness) and sensory component (worst pain) (Pagé et al., 2012), fun and nausea (Hoffman et al., 2004a) were evaluated in both conditions (“No VR” vs. “Yes VR”, within subjects). The quality of VR experience was investigated only in the “Yes VR” condition asking patients what extent did they feel like they went into the virtual world, and how real did the VR objects seem. The included questions were based on those used in previous studies (Hoffman et al., 2004a; Atzori et al., 2018b) and they were translated from English language into Italian language using the back-translation method, one of the most commonly used methods for cross-cultural translation (Maneesriwongul and Dixon, 2004). The total time for the procedure was comparable in both conditions. The time was measured from the positioning of the tourniquet to the needle extraction.

### Immersive VR System

The VR equipment consisted of a VR helmet, the Personal 3D Viewer Sony: HMZ T-2, supported by a laptop, that allowed the interaction with the VR environment. The helmet had a 45° diagonal field of view, 1280 x 720 pixels per eye, latex-free earphones to provide acoustic isolation and it was suitable for both younger and older children. The VR software used was Snow World^2^, one of the most frequently employed VR environments, specifically designed to promote distraction from procedural pain (Hoffman et al., 2001). In SnowWorld, patients “go into” an icy canyon, where they throw snowballs at penguins, snowmen and other characters in VR, using a wireless mouse with the hand not employed in the venipuncture. SnowWorld was previously used in studies evaluating VR effectiveness for pain reduction in burn patients and during dental procedures. This is the first study in which this virtual environment is applied during venipuncture in patients with oncological and blood diseases.

### Data Analysis

A *t*-test for paired samples was adopted to compare pain, nausea and fun levels and the total time for the procedure between the “No VR” condition and the “Yes VR” condition. A researcher not involved in data collection carried out data analysis using the statistical Software SPSS. Based on the *a priori* assumption that the differences could only be in one direction, results were considered significant when associated with *p*-values less than 0.05, one tailed.

## Results

### Pain

As reported in Table 2, when patients underwent venipuncture, their mean pain levels were significantly lower during VR, compared with pain levels during “No VR”, for all the three pain components: “*Time spent thinking about pain*” during “No VR” mean = 3.23 (*SD* = 2.98) vs. during “Yes VR” mean = 1.33 (*SD* = 1.05), *p* < 0.05, Cohen’s *d* = 0.62, moderate effect size; *“Pain unpleasantness*” during “No VR” mean = 3.27 (*SD* = 3.43) vs. during “Yes VR” mean = 0.93 (*SD* = 1.16), *p* < 0.01, Cohen’s *d* = 0.70 moderate effect size; *“Worst pain*” during “No VR” mean = 3.60 (*SD* = 3.00) vs. during “Yes VR” mean = 2.00 (*SD* = 1.20), *p* < 0.05, Cohen’s *d* = 0.51, moderate effect size.

**Table 2:** Means (Standard Deviation) in “No-VR” condition vs. “Yes-VR” condition.

	No-VR Mean (SD)	Yes-VR Mean (SD)	*t* (df)	*p*-value (Sig 1-tail)	Cohen’s d Effect size
Time spent thinking about pain	3.23 (2.98)	1.33 (1.05)	2.39 (14)	< 0.05	.62 moderate
Worst Pain	3.60 (3.00)	2.00 (1.20)	1.99 (14)	< 0.05	.51 moderate
Pain Unpleasantness	3.27 (3.43)	0.93 (1.16)	2.70 (14)	< 0.01	.70 moderate
Nausea	0.80 (2.60)	0.00 (0.000)	1.19 (14)	0.25 NS	
Fun	2.93 (3.58)	8.80 (1.42)	−6.60 (14)	< 0.001	1.71 large effect size
Presence		7.93 (1.79)			
Realism of VR objects		6.80 (2.37)			

### Quality of VR Experience, Fun, Nausea and Total Time for the Procedure

Patients distracted by VR reported a mean presence score of 7.93 (*SD* = 1.79), corresponding to “strong sense of going inside the computer generated world”, and a mean realism of VR objects score of 6.80 (*SD* = 2.37), corresponding to “Moderately real.”

Patients rated “fun” during the venipuncture as “mildly fun” during No VR, vs. “pretty fun” during VR. A significant difference for fun levels emerged between the two conditions: during “No VR” mean = 2.93 (*SD* = 3.58) vs. “Yes VR” mean = 8.80, *SD* = 1.42; *t*(14) = −6.60, *p* < 0.0001, Cohen’s *d* = 1.71, large effect size. No significant differences emerged for nausea levels between the two conditions (*p* > 0.05 NS): no patient reported nausea during the interaction with VR. During the “Yes VR” condition the mean of the total time of the procedure was 3.09 min (*SD* = 1.81) vs. 4.45 (*SD* = 3.50) during the “No VR” condition. The pattern of results showed the venipuncture took less time during VR vs. during No VR; however, the differences were not significant (*p* > 0.05, NS).

### Gender Effects

**FIGURE 1:**
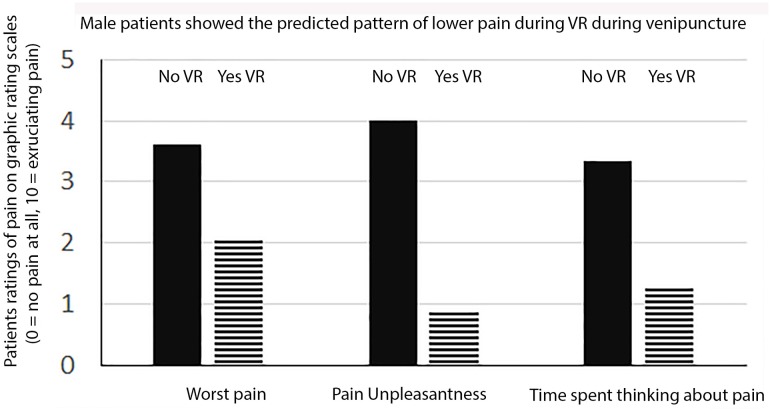
Male patients ratings of pain.

**FIGURE 2:**
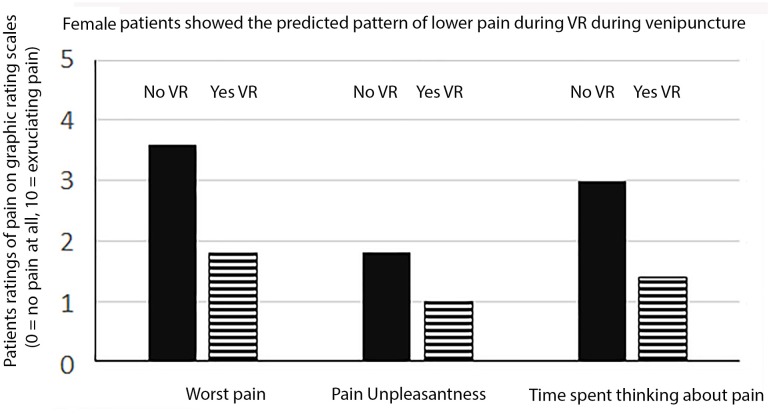
Female patients ratings of pain.

As shown in Figures 1, 2 when males and females were analyzed separately, both males and females showed the predicted pattern of results (lower pain during VR compared to standard of care No VR).

## Discussion

Based on the Eccleston and Crombez (1999) Interruption of Attention and Pain model, pain requires attention, and humans have limited attentional resources, we predicted that patients would focus their attention on VR, and would have fewer attentional resources available to focus on the painful stimulus, so patients would feel less pain. As predicted, children and adolescent patients reported significant reductions in pain unpleasantness, reported spending significantly less time thinking about their pain during venipuncture and reported significantly lower intensity of pain during VR. As predicted, children and adolescent patients reported significantly more fun when they used VR during their venipuncture. In the current study, patients experienced a strong illusion of presence and rated the virtual objects as “moderately real” looking. According to these results, the current VR system including the Sony HMZ-T2 helmet and the VR software SnowWorld, could be considered suitable for clinical applications with children and adolescents, promoting a medium-high quality virtual experience, without side effects. A higher quality helmet could potentially promote even better analgesia, as suggested by the literature (Hoffman et al., 2006).

The current study has some important limitations. Firstly, the sample size is small. Future studies with a larger sample are needed, and should also evaluate how much VR distraction reduces anxiety. Another limitation of the current study is the use of standard of care as a control group. Because the current study used standard of care as the control group, the difference between the groups may simply be due to the use of a distraction technique rather than to the specific use of VR. Addition research comparing VR to a more conventional distraction technique such as listening to music, is needed before any firm conclusions can about whether VR is unusually distracting.

According to Gold et al. (2007), not only the attentional demanding, but also the elicitation of positive emotion may contribute to VR analgesia (e.g., Sharar et al., 2016). The isolation from the medical setting (helmet blocking the patients view of the hospital room) and the possibility to be immersed in a pleasant activity makes VR a strong distraction technique, in particular for younger patients. The current study compared VR distraction with treatment as usual (non-medical conversation).

Future studies should compare VR to other distraction techniques during venipuncture, and should further explore the role of emotional activation in VR analgesia. For example, in future studies the VR condition should be compared to another simpler form of distraction such as having the children listen to an audiotape while undergoing the venipuncture treatment. And a study comparing immersive VR to augmented reality glassescould be interesting. Moreover, patients interacted with Snow World, a virtual environment specifically designed for procedural pain management, in particular for burn patients. In future studies, environments designed for the specific kind of procedure and patient’s characteristics (i.e., age, gender, cognitive abilities) are recommended.

Future studies may explore whether personality aspects of the child, such as catastrophizing, fear of pain, as well as parents’ anxiety, and patients’ memory for previous painful procedures (Noel et al., 2015) influence how much pain children experience during medical procedures (e.g., De Castro Morais Machado et al., 2018).

## Conclusion

This study contributes to a growing literature that supports the use of immersive VR distraction for pain control. The current study evaluated the effectiveness of VR to control pain (in its affective, sensory and cognitive components) and to promote fun during venipuncture in pediatric patients with cancer and blood diseases. Younger patients suffering from chronic diseases (i.e., cancer and blood diseases), who spend much time in hospital and need several painful and stressful medical procedures, could particularly benefit from this distraction. In the future, VR systems could also let patients have social interaction (Won et al., 2017) and the quality of VR experiences will be more and more attentional demanding. VR distraction may also offer new opportunities for socialization and social support, especially for those patients in isolation or hospitalized for long periods.

## Author Contributions

All authors listed have made a substantial, direct and intellectual contribution to the work, and approved it for publication.

## Conflict of Interest Statement

The authors declare that the research was conducted in the absence of any commercial or financial relationships that could be construed as a potential conflict of interest.

## Footnotes

1 www.wma.net

2 http://www.vrpain.com
